# Metabolomics analysis of anaphylactoid reactions induced by Xueshuantong injection in normal and immunocompromised mice

**DOI:** 10.3389/fphar.2024.1526875

**Published:** 2025-01-06

**Authors:** Xiaoqian Guo, Chi Zhang, Yingyu Li, Wen Wen, Yinghui He, Feng Tang, Chunming Chen, Chao Hu, Linqi OuYang, Wenlong Liu, Zhenhua Zhu, Hongyu Liu

**Affiliations:** ^1^ The First Hospital of Hunan University of Chinese Medicine, Changsha, China; ^2^ Hunan Key Laboratory of Druggability and Preparation Modification of Traditional Chinese Medicine, Changsha, China; ^3^ Hunan Industry and Commerce Career Academy, Hengyang, China; ^4^ Changsha Hospital of Traditional Chinese Medicine, Changsha, China; ^5^ College of Pharmacy, Hunan University of Chinese Medicine, Changsha, China

**Keywords:** anaphylactoid reaction, different immunization status, Xueshuantong injection, metabolic pathway, metabolomics

## Abstract

**Background:**

Xueshuantong injection (Lyophilized) (XSTI) is widely used to treat cardiovascular and cerebrovascular diseases. However, anaphylactoid reactions (ARs) are frequently reported as one of its side effects, and the mechanisms of ARs and their relationship with the different immune status are still not well understood.

**Purpose:**

This article aims to examine the sensitizing effect of XSTI, explore the impact of normal and immunocompromised states on ARs, and analyze AR-related metabolic pathways by metabolomics.

**Methods:**

An immunocompromised mouse model was established through intraperitoneal injection of cyclophosphamide (CTX). Normal and immunocompromised mice were then treated with normal saline (NS), histamine (HIS), and XSTI, respectively. Behavioral responses, auricle blue staining, and Evans blue (EB) exudation were used as indices to evaluate the sensitization of XSTI on both normal and immunocompromised mice. Subsequently, ARs models with different immune statuses were established, and validated by measuring four serum indicators using enzyme-linked immunosorbent assay (ELISA). Finally, LC-MS metabolomics analysis was performed on mouse serum to evaluate the metabolic pathways.

**Results:**

The intensity of ARs induced by XSTI in mice was found to increase with the administered dose, with normal mice exhibiting higher AR intensities compared to immunocompromised mice. Metabolomic analysis revealed significant metabolic changes in XSTI-treated mice. The metabolic pathways predicted from these different metabolites include biotin metabolism, histidine metabolism, glycerolipid metabolism, bile secretion, arachidonic acid metabolism, sphingolipid metabolism, niacin and nicotinamide metabolism, tryptophan metabolism, steroid biosynthesis, and arginine and proline metabolism.

**Conclusion:**

Research indicated that the sensitization of XSTI is dose-dependent, and mice with weakened immune functions exhibit lower sensitivity. Through metabolomics research, the differential metabolites in mice were analyzed, and the metabolic pathways inducing ARs were predicted. This study offers guidance on safe medication from the perspective of organism susceptibility and lays a foundation for research on the potential mechanisms of ARs.

## Graphical Abstract

**Figure:**
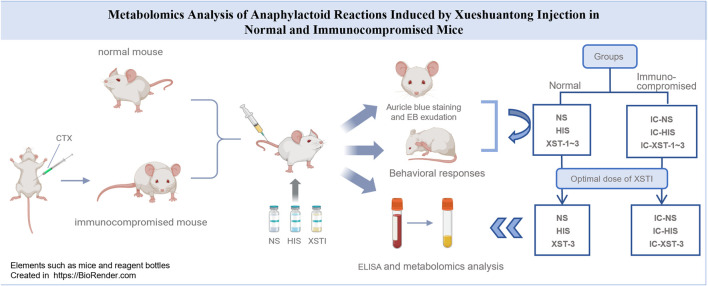

## 1 Introduction

Anaphylactoid reactions (ARs) are the most common adverse reactions of traditional Chinese medicine injections (TCMIs), accounting for 77% of total adverse reactions (ADRs) (Yang, Y. et al., 2019). The symptoms associated with ARs are akin to those observed in classic anaphylaxis reactions, including skin rashes, itching, redness, palpitations, and shortness of breath (Chen, M. et al., 2017). However, the difference lies in that ARs do not depend on IgE-mediated immune activation and occur after the first administration of the drug (Fan, X. et al., 2018; Cianferoni, 2021). It is currently hypothesized that the mechanism of ARs involves direct or indirect action of drug components on mast cell (MC) and basophilic granulocytes, leading to the release of active mediators, possibly through activation of the complement system and other pathways (Li, L. et al., 2012; Yang, Y. et al., 2019). Further research is needed to elucidate the specific mechanisms involved. Currently, research on ARs primarily focuses on animal experiments in normal physiological states, aiming to identify sensitizing substances and conduct mechanism studies. There is limited research involving animals in different physiological states (Xie, J. et al., 2019; Xiao et al., 2020).

Metabolomics, as a systematic approach, has emerged as a crucial tool for elucidating the pharmacodynamic mechanisms of TCMIs. It allows for the characterization of the overall metabolic status of the organism and can be utilized to enhance the study of TCMI-induced ARs in different physiological states of the organism (Wei et al., 2011; Ma and Qi, 2021). By employing metabolomics, potential biomarkers can be identified, and their mechanisms can be systematically revealed, contributing to a deeper understanding of TCMI-induced ARs.

Xueshuantong injection (XSTI) (lyophilized) is an injectable solution containing Panax notoginseng saponins as its main active components, which are extracted from the Araliaceae plant *Panax notoginseng* (Burk.) F. H. Chen, and primarily consist of notoginsenoside R_1_, along with ginsenoside Rd, Rg_1_, and Rb_1_. Studies have demonstrated that XSTI possesses anti-myocardial infarction, anti-thrombosis, anti-cerebral ischemia, anti-oxidation, and improving microcirculatory disorders, making it widely used in the treatment of cardiovascular and cerebrovascular diseases (Li C. et al., 2018; Wang et al., 2022). However, the increasing reports of ADRs about XSTI, primarily ARs, have limited its clinical application (Zhou et al., 2014; Shen and Zhou, 2015).

In this research, we selected hyper-aphylactic ICR mice as model animals and manipulated their immune status. Based on this manipulation, XSTI was utilized as the model drug to establish a model of anaphylactoid reaction in mice with varying immune statuses. We aim to compare the susceptibility and intensity of drug-induced ARs in organisms under normal and immunocompromised states, to explore the impact of different immune states on ARs.

## 2 Materials and methods

### 2.1 Reagents and materials

Xueshuantong injection (lyophilized) was purchased from Guangxi Wuzhou Pharmaceutical (Group) Co., Ltd. (Guangxi, China) (Batch number: 20100113). This product primarily contains five compounds extracted from the *Panax notoginseng* (Burk.) F. H. Chen [Araliaceae; Notoginseng Radix et Rhizoma]: notoginsenoside R_1_ (9.5%–15.0%), ginsenoside Rg_1_ (37.0%–56.0%), ginsenoside Re (5.0%–8.0%), ginsenoside Rb_1_ (21.0%–32.0%), and ginsenoside Rd (greater than 0.45%). The certificate of analysis of XSTI is shown in Supplementary. Panax notoginseng (Burk.) F.H. Chen, a species in the genus Panax (Family Araliaceae), has its taxonomic classification confirmed by the MPNS (http://mpns.kew.org/mpns-portal/). Mouse ELISA Kit was purchased from Shanghai Fusheng Industry Co., Ltd. (Shanghai, China). Normal saline (NS) was purchased from Hunan Kelun Pharmaceutical Co., Ltd. (Yueyang, Hunan, China) (Batch number: G21050509). Histamine (HIS) was purchased from Shanghai McLean Biochemistry Science and Technology Co. (Shanghai, China) (Batch number: C12812525). Evans Blue was purchased from Shanghai McLean Biochemical Science and Technology Co., Ltd. (Shanghai, China) (Batch number: C1069175). Formamide was purchased from Shanghai McLean Biochemical Science and Technology Co., Ltd. (Shanghai, China) (Batch number: C13106895). Andosan (cyclophosphamide) was purchased from Baxter Oncology GmbH. (Germany) (Lot number: H20160467).

### 2.2 Drug preparation

According to the human dosing guidelines of XSTI (maximum daily dose of 5 mg/kg for a 60 kg adult), the standard dosage for mice was determined using a body surface area conversion method. The low, medium, and high dose groups for XSTI were then established at 2-fold, 4-fold, and 8-fold of the standard dose. NS was used as the solvent to prepare 0.25 g/kg HIS solution, 0.08 g/kg cyclophosphamide (CTX) solution, and XSTI at three concentration levels (0.044 g/kg, 0.088 g/kg, 0.176 g/kg). Additionally, NS, HIS, and XSTI were prepared as injections containing 0.8% Evans Blue (EB).

### 2.3 Animals

SPF male ICR mice (25–30 g) were obtained from Slake Jingda Experimental Animal Co., Ltd. (Hunan, China). The mice were kept under controlled conditions, with temperatures maintained at 20°C–26°C and humidity levels at 40%–70%, within the barrier facility of the Experimental Animal Center at Hunan University of Chinese Medicine (Changsha, China) and acclimatized for 3–5 days. Experimental procedures received approval from the Institutional Animal Ethics Committee (License number: SYXK 2019–0009; animal ethics number: LLBH-202111030001) and were carried out in compliance with the established institutional animal care guidelines and protocols.

### 2.4 Establishment and validation of immunocompromised mouse model

The immunocompromised mouse model was established by intraperitoneal injection of CTX. ICR mice were randomly assigned to six groups (*n* = 8): three control groups (Con-1, Con-2, Con-3), with no treatment, and three experimental groups (Model-1, Model-2, Model-3) receiving intraperitoneal injections of CTX (0.08 g/kg) for three consecutive days, followed by a five-day adaptive feeding period. The experimental groups were designed to evaluate different aspects of immune function: nonspecific immunity (Model-1), humoral immunity (Model-2), and cellular immunity (Model-3). The body weight growth ratio of each mouse was calculated using the formula: (final body weight - initial body weight)/initial body weight.

#### 2.4.1 Carbon clearance

The carbon clearance index (*K*) and the phagocytic index (α) were used to assess macrophage function, thereby reflecting the strength of nonspecific immune capabilities (Wang et al., 2011; Li Q. et al., 2018). In the Model-1 group experiment, 25% India ink was injected intravenously at a dosage of 0.01 mL/g. Blood samples were drawn from the inner canthal venous plexus at 2 min and 10 min post-injection. The optical density (OD) value was measured at 630 nm using ELISA to calculate the carbon clearance index and the phagocytic index. Finally, the mice were euthanized by cervical dislocation, and their livers, spleens, and thymuses were collected and weighed. The calculation formula is as follows:

$$\alpha =\frac{body\;weight}{liver\;weight+spleen\;weight}\sqrt[{3}]{K}$$

$$K=\frac{\mathrm{lg}A_{1}-\mathrm{lg}A_{2}}{t_{2}-t_{1}}$$

α: phagocytosis index, *K*: carbon profile index, A_1_: OD value (2 min), A_2_: OD value (10 min), t_1_: 2 min, t_2_: 10 min.

#### 2.4.2 Spleen and thymus indices

The spleens and thymuses, key immune organs, were used to evaluate the immune function of the mice (Wang Y. et al., 2016). The spleen and thymus indices were calculated using the formulas: Spleen index = spleen weight (mg)/body weight (g), and Thymus index = thymus weight (mg)/body weight (g).

#### 2.4.3 Serum hemolysin

Serum hemolysin levels were used to evaluate alterations in the humoral immunity of mice (Zhao et al., 2014; Li Q. et al., 2018). On the first day of modeling, mice in the Model-2 and Con-2 groups received an intraperitoneal injection of 0.2 mL of a 5% chicken red blood cell suspension. 30 min later, the Model-2 group received an intraperitoneal injection of CTX. The day after modeling, blood was collected from the ocular veins of mice in both groups. The collected blood was centrifuged at 3,000 rpm for 10 min, and the supernatant serum was diluted 1:100 with NS. 1 mL of diluted serum was combined with 0.5 mL of 5% chicken red blood cell suspension and 0.5 mL of 10% guinea pig serum (complement). The mixture was incubated at 37°C for 30 min, then cooled to 0°C to stop the reaction. After a second centrifugation at 3,000 rpm for 10 min, 200 µL of the supernatant was transferred to a 96-well plate. The OD value at 570 nm was measured using ELISA to determine serum hemolysin levels.

#### 2.4.4 Delayed-type hypersensitivity

Delayed-type hypersensitivity (DTH), a T cell-mediated immune response, serves as a measure of cellular immune function in mice (Makare et al., 2001; Wang et al., 2017). On the third day of modeling, the abdominal area of the Model-3 group, approximately 3 cm × 3 cm in size, was depilated and uniformly applied with 50 µL of a 1-fluoro-2,4-dinitrobenzene (DNFB) solution daily for 2 days. On the fourth day, 20 µL of DNFB solution was applied to the right ear of mice in both the Model-3 and Con-3 groups to elicit DTH. 24 h later, the mice were euthanized, and their ears were collected. Ear punches with an 8 mm diameter were excised and weighed. The degree of swelling in the mice’s ears (swelling degree = right ear mass - left ear mass) was used to assess the intensity of DTH.

### 2.5 ARs in mice with different immune states

Normal mice were randomly divided into five groups (n = 8). Group 1 (NS) was the control group and received NS. Group 2 (HIS), the positive control group, received HIS solution (0.25 g/kg). Groups 3 to 5 (XST-1, XST-2, XST-3) received low, medium, and high doses of XSTI (0.044 g/kg, 0.088 g/kg, 0.176 g/kg), respectively. Immunocompromised mice were also divided into five groups: IC-NS, IC-HIS, IC-XST-1, IC-XST-2, and IC-XST-3. Each group was injected with the respective drug (containing 0.8% Evans Blue) via the tail vein.

After administration, the symptoms of the mice were observed within 30 min. Based on the criteria for evaluating AR symptoms (Tables 1, 2) (Xie, J. et al., 2019), the intensity of the ARs in mice was assessed. Afterward, the mice were euthanized, and their ears were collected.

**TABLE 1:** Scoring criteria of ARs symptoms.

Symptomatic behavior	Score
Normal activity	0
Restlessness, bristling, decreased activity, trembling, frequent nose or ear scratching	1
Shortness of breath, swollen mouth and nose, swelling, tearing, cyanosis	2
Dyspnea, unsteady gait, jumping, spasms, spinning	3
Death	4

**TABLE 2:** Intensity levels of ARs.

Intensity coefficient of ARs (K)	Level
0	negatives
>0, ≤1	weak positive
>1, ≤2	positive
>2, ≤3	Strong Positive
>3	Extremely Strong Positive

The staining area was scored as per Table 3. Chop the auricle into pieces, soak in 2 mL of formamide, and store in the dark at room temperature for 48 h. In formamide as a solvent, EB was prepared into a working solution of 10 μg/mL and subsequently diluted to concentrations of 0.5, 0.25, 0.2, 0.1, 0.05, 0.025, 0.005, and 0 mg/L. The OD values at 630 nm were measured by ELISA to establish a standard curve. Extracts from the incubated ears were centrifuged at 3,000 rpm for 15 min, and the supernatant was collected to measure the OD value at 610 nm.

**TABLE 3:** Auricle blue staining scoring criteria.

Auricular blue-stained area (S)	Score
0 (no blue dye)	0
0<S≤1/8	1
1/8<S≤1/4	2
1/4<S≤1/2	3
1/2<S≤3/4	4

### 2.6 Establishment and validation of ARs model

The ARs model was established using the optimal sensitizing dose of XSTI. Mice were randomly divided into two main groups: normal and immunocompromised. The normal group was subdivided into three subgroups: blank control (NS), positive control (HIS), and XSTI treatment (XST-3). Similarly, the immunocompromised group, induced by CTX, was divided into the corresponding subgroups: IC-NS, IC-HIS, and IC-XST-3. In total, six groups of twelve mice were established. To explore the relationship between AR intensity and response time, the groups were further subdivided based on blood collection times: 10 min and 30 min post-administration. Blood samples were collected from the ocular veins at these intervals, centrifuged at 3,000 rpm for 15 min, followed by a secondary centrifugation at 12,000 rpm for 10 min, and then cryopreserved at −80°C. Samples were analyzed using ELISA and metabolomics techniques. ELISA was used to measure serum levels of tryptase (TPS), β-hexosaminidase (β-Hex), immunoglobulin E (IgE), and HIS to validate the model. The ARs intensity at various drug dosages and time points was assessed by analyzing the serum concentrations of TPS, β-Hex, IgE, and HIS, following the ELISA kit instructions.

### 2.7 LC-MS sample preparation

Each serum sample (100 µL) was mixed with a fourfold volume of a methanol/acetonitrile (1:1, v/v) mixture, vortexed for 30 s, and then sonicated in an ice-water bath for 10 min. Subsequently, the samples were left to stand for 1 h at −40°C, followed by centrifugation at 12,000 rpm for 15 min at 4°C. Quality control (QC) samples were prepared in equal amounts using the same method.

### 2.8 LC-MS analysis parameters

Separation was performed using the Vanquish Ultra High-Performance Liquid Chromatograph (UPLC) (Thermo Fisher Scientific, United States) paired with a Waters ACQUITY UPLC BEH Amide column (2.1 mm × 100 mm, 1.7 µm) (Wang J. et al., 2016). The sample tray was kept at 4°C with an injection volume set to 2 µL. Mobile phase A comprised 0.1% ammonium acetate (25 mmol/L) and ammonia (25 mmol/L) in water, while mobile phase B was acetonitrile. Data acquisition for primary and secondary mass spectrometry was controlled using Xcalibur software on Thermo Q Exactive HFX mass spectrometer (Thermo Fisher Scientific, United States). The settings were: sheath gas flow rate at 30 Arb, auxiliary gas flow rate at 25 Arb, capillary temperature at 350°C, full MS resolution at 120,000, MS/MS resolution at 7,500, and spray voltage at 3,600 V in positive mode or −3600 V in negative mode.

### 2.9 Metabolomics data processing and analysis

The raw data were initially converted to mzXML format using ProteoWizard software. Peak identification, extraction, alignment, and integration were subsequently carried out with a custom-written R package with XCMS as the core (Dunn et al., 2011). Processed data were annotated by matching against a self-built secondary mass spectrometry database in BiotreeDB (version 2.1), with the cutoff value for algorithm scoring set at 0.3. Deviant values were filtered to remove noise from individual peaks based on the coefficient of variation (CV). Missing values were addressed by filtering individual peaks, retaining only those with no more than 50% missing values in any single group or across all groups. Missing peak area data were imputed using half the minimum observed value. Finally, data normalization was conducted using internal standards (Kuroishi, 2015).

Data were log-transformed and centered using SIMCA software before automated modeling analysis (Rodriguez-Melendez and Zempleni, 2003). The first principal component identified was then analyzed using the OPLS-DA method. Model validity was assessed by combining univariate and multivariate statistical analyses with a 7-fold cross-validation, and further validated using permutation tests. Differential metabolites were identified based on a Variable Importance in the Projection (VIP) greater than 1 for the first principal component of OPLS-DA, and the Student’s t-test *p*-value less than 0.05.

### 2.10 Statistical analysis

Statistical analysis of various indices in mice was conducted using GraphPad Prism 8.0, with results expressed as mean ± standard deviation. For non-normally distributed data, Tamhane’s one-way ANOVA in SPSS21 was applied for comparisons. A *p*-value of less than 0.05 was deemed statistically significant.

## 3 Results

### 3.1 Validation of immunocompromised models

**FIGURE 1:**
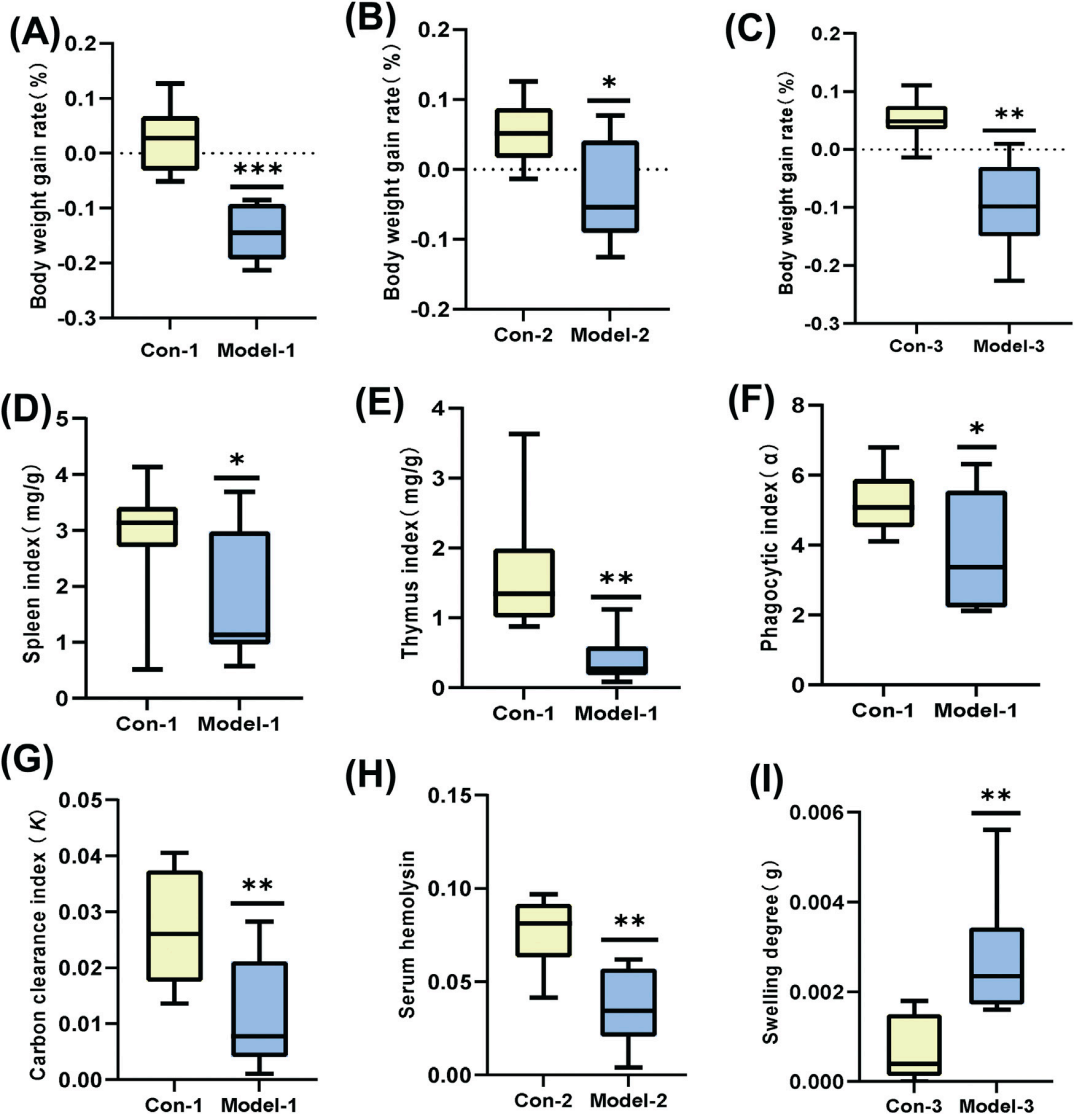
**(A–C)**: Growth rate of body weight. **(D, E)**: Mouse spleen index and thymus index. **(F, G)**: Mouse phagocytic index (α) and carbon clearance index (*K*). **(H)**: Mouse serum hemolysin levels. **(I)**: Mouse-ear swelling degree. (*p < 0.05, **p < 0.01).

Among multiple test indicators (Figure 1), the weight gain rate of the modeled mice was slower compared to normal mice. The spleen and thymus indices decreased significantly (*p* < 0.05 and *p* < 0.01, respectively), indicating notable suppression of immune function. Both the phagocytic index α and the clearance index K were reduced (*p* < 0.05 and *p* < 0.01, respectively), suggesting a substantial weakening of non-specific immune function. The serum hemolysin content also decreased (*p* < 0.01), reflecting a significant decline in humoral immune function. Furthermore, the degree of ear swelling increased (*p* < 0.01), indicating an enhanced DTH reaction and a pronounced reduction in cellular immune function. Consequently, we successfully established an immunocompromised mouse model.

### 3.2 ARs in mice with varying immune states

#### 3.2.1 Behavioral analysis

**FIGURE 2:**
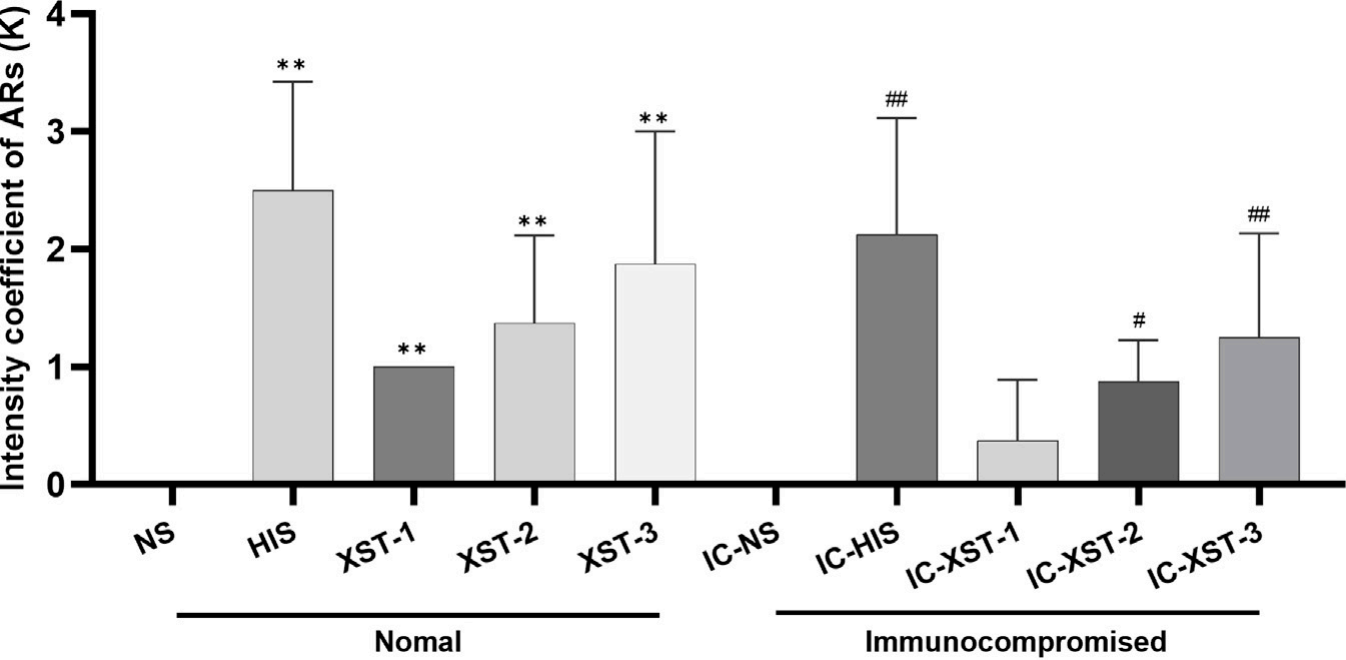
Intensity coefficient of ARs (K).

Firstly, the behavior of mice post-medication was observed, and the ARs strength coefficient K for each group was calculated (Figure 2). Significant differences were found between the HIS, XST-1, XST-2, XST-3 groups and the NS group (*p* < 0.01). Likewise, the IC-HIS (*p* < 0.01), IC-XST-2 (*p* < 0.05), and IC-XST-3 (*p* < 0.01) groups showed significant differences compared to the IC-NS group. The K value reflects the AR intensity level in mice (Table 2), with negative responses in NS treatment groups, strong positive in HIS treatment groups, weak positive in the XST-1, IC-XST-1, and IC-XST-2 groups, and positive in the XST-2, XST-3, and IC-XST-3 groups.

#### 3.2.2 Auricular blue staining and EB exudation

**FIGURE 3:**
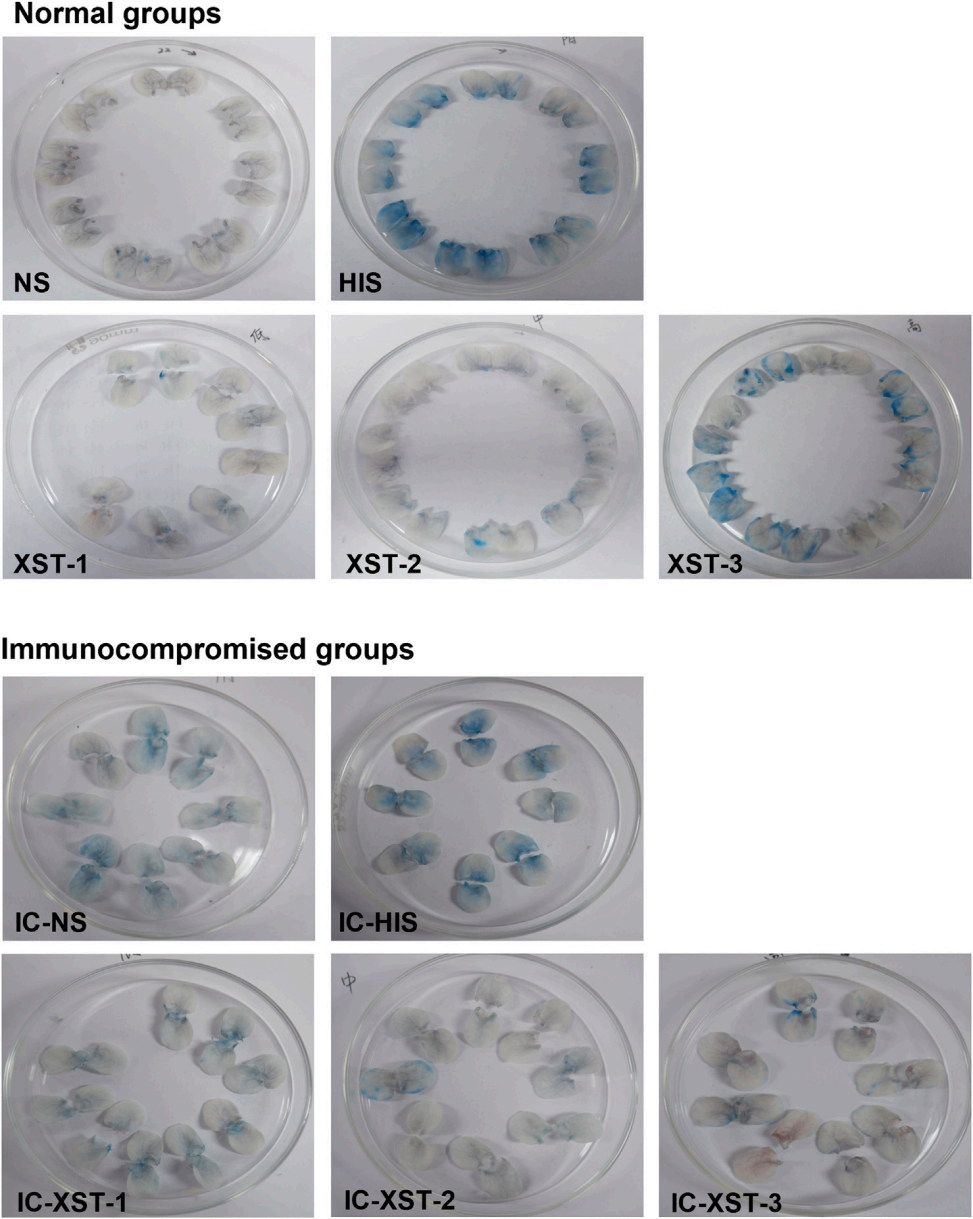
Auricle Blue staining.

**FIGURE 4:**
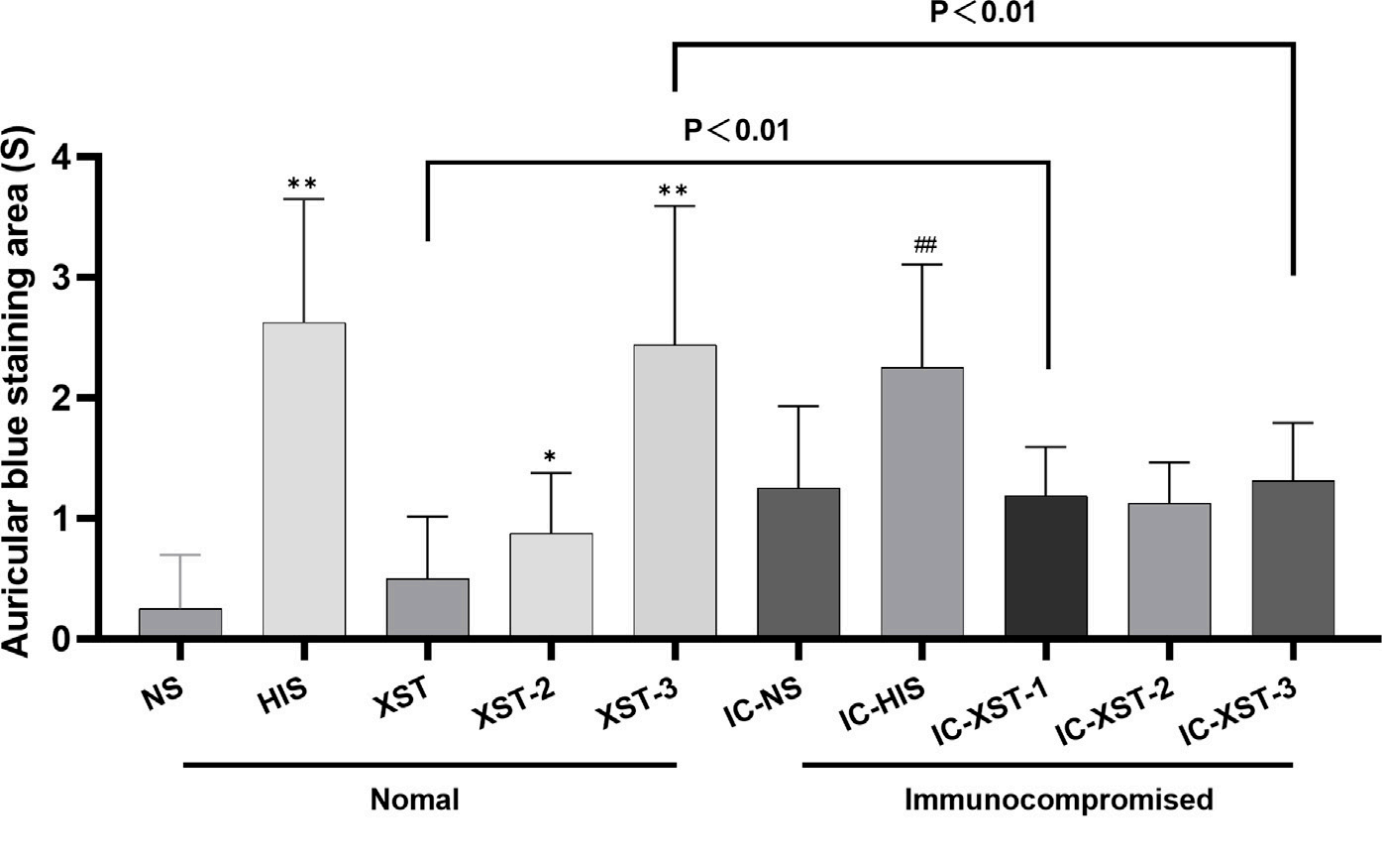
Auricular Blue staining area (S). (Compared with NS group, **p < 0.01, *p < 0.05. Compared with IC-NS group, ##*p* < 0.01).

**FIGURE 5:**
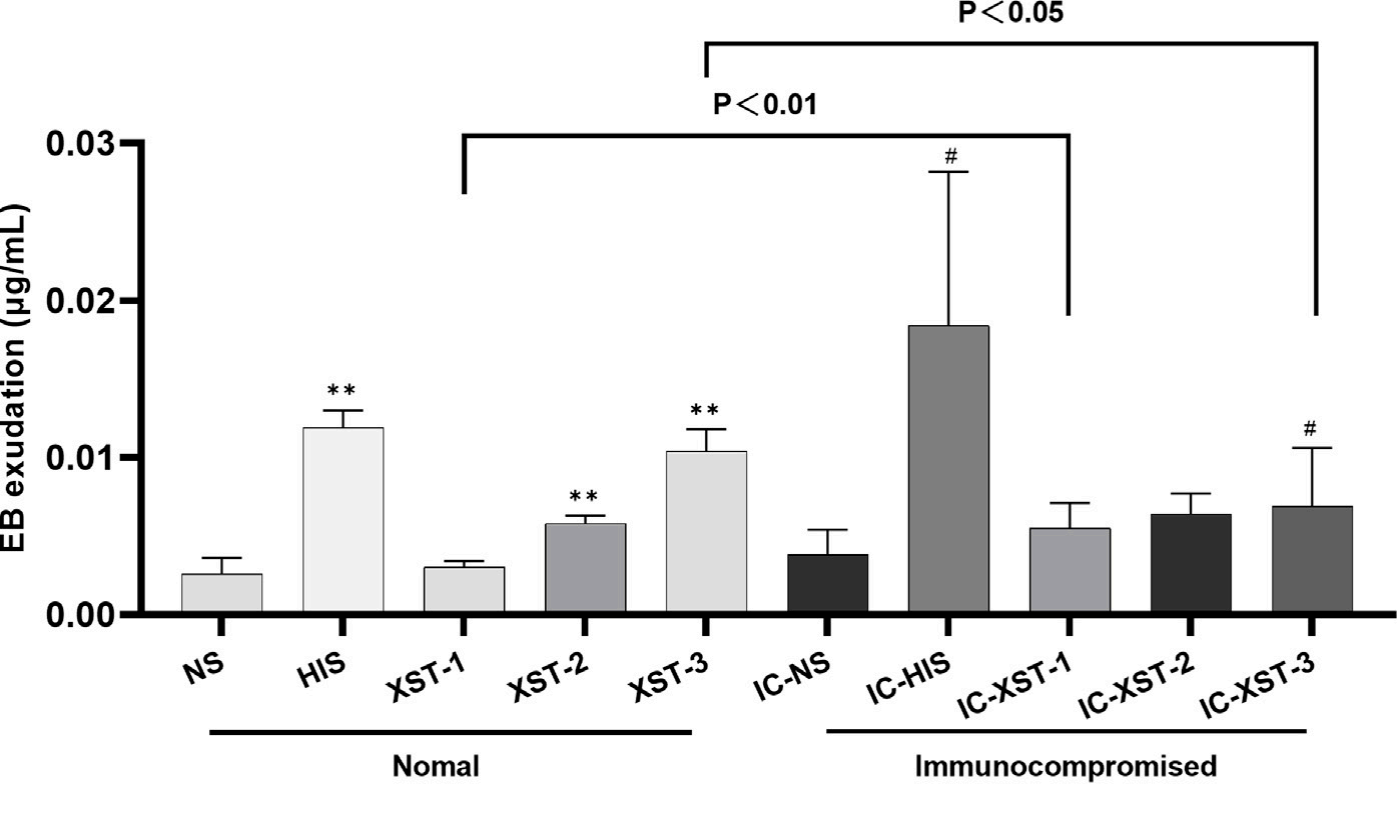
EB exudation. (Compared with NS group, **p < 0.01. Compared with IC-NS group, #*p* < 0.05).

Auricular blue staining and EB exudation assay to detect increased vascular permeability in mouse ears caused by ARs. In normal mice, the auricular blue staining area expanded with increasing XSTI dosage (Figures 3, 4). The EB exudation concentration was determined using the EB standard curve (Figure 5). Compared to the NS group, the HIS, XST-2, and XST-3 groups exhibited significantly higher concentrations (*p* < 0.01). Similarly, the IC-HIS, IC-XST-2, and IC-XST-3 groups showed increased concentrations compared to the IC-NS group (*p* < 0.05). In comparison of auricular blue staining area in mice with different immune statuses under the same drug, XST-1 group was lower than IC-XST-1 group (*p* < 0.01), and XST-3 group was higher than IC-XST-3 group (p < 0.01); EB extravasation was compared, XST-1 group was lower than IC-XST-1 group (*p* < 0.01), and XST-3 group was higher than IC-XST-3 group (*p* < 0.05).

#### 3.2.3 Optimal sensitizing dose of XSTI

The optimal XSTI dosage was determined by assessing its sensitization in normal mice, using the ARs strength coefficient (K), auricular blue staining area, and EB exudation as key indicators. The results showed that sensitization increased with higher XSTI doses, with the maximum dose (0.176 g/kg) identified as optimal for establishing the ARs model.

### 3.3 ARs model validation

**FIGURE 6:**
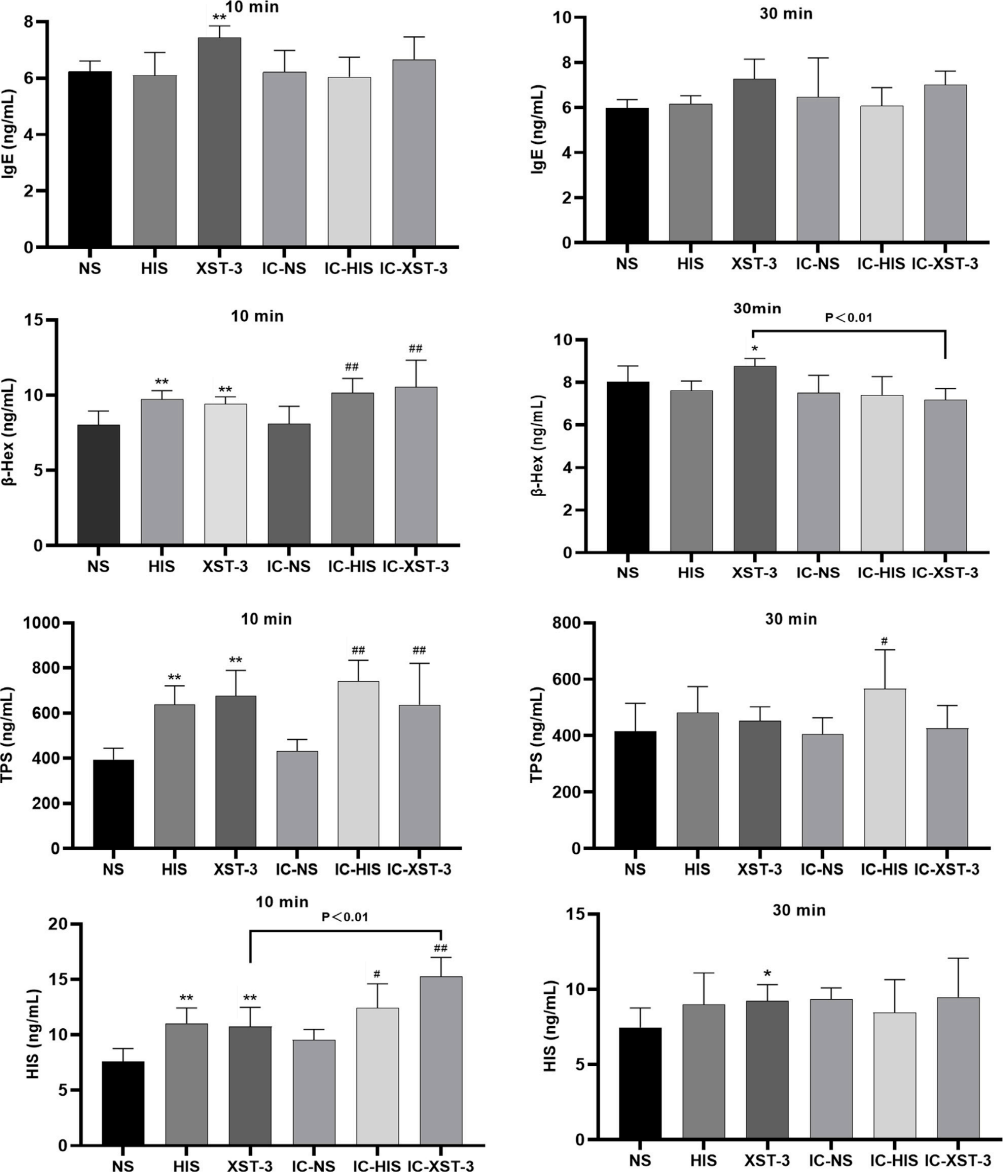
Concentrations of IgE, β-Hex, TPS, and HIS in the serum at 10 min and 30 min respectively. (*p < 0.05, **p < 0.01 compared to the NS group; #*p* < 0.05, ##*p* < 0.01 compared to the IC-NS group).

The serum indicators were analyzed using ELISA (Figure 6). In normal mice groups, 10 min post-administration, TPS, β-Hex, and IgE concentrations in the HIS and XST groups were significantly higher than those in the NS group (*p* < 0.01). After 30 min, the β-Hex concentration in the XST group remained elevated (*p* < 0.05). In immunocompromised mice, 10 min post-administration, TPS and β-Hex concentrations in the IC-HIS and IC-XST-3 groups were significantly higher than those in the IC-NS group (*p* < 0.01). After 30 min, the TPS concentration in the IC-HIS group remained elevated (*p* < 0.05). Comparing the normal and immunocompromised groups, the β-Hex concentration of the XST group was lower than in the IC-XST-3 group at 10 min (*p* < 0.01), and the HIS concentration in the XST group was lower than in the IC-XST-3 group at 30 min (*p* < 0.01).

The increase in these indicator concentrations confirms the successful establishment of the ARs model.

### 3.4 LC-MS stability analysis

**FIGURE 7:**
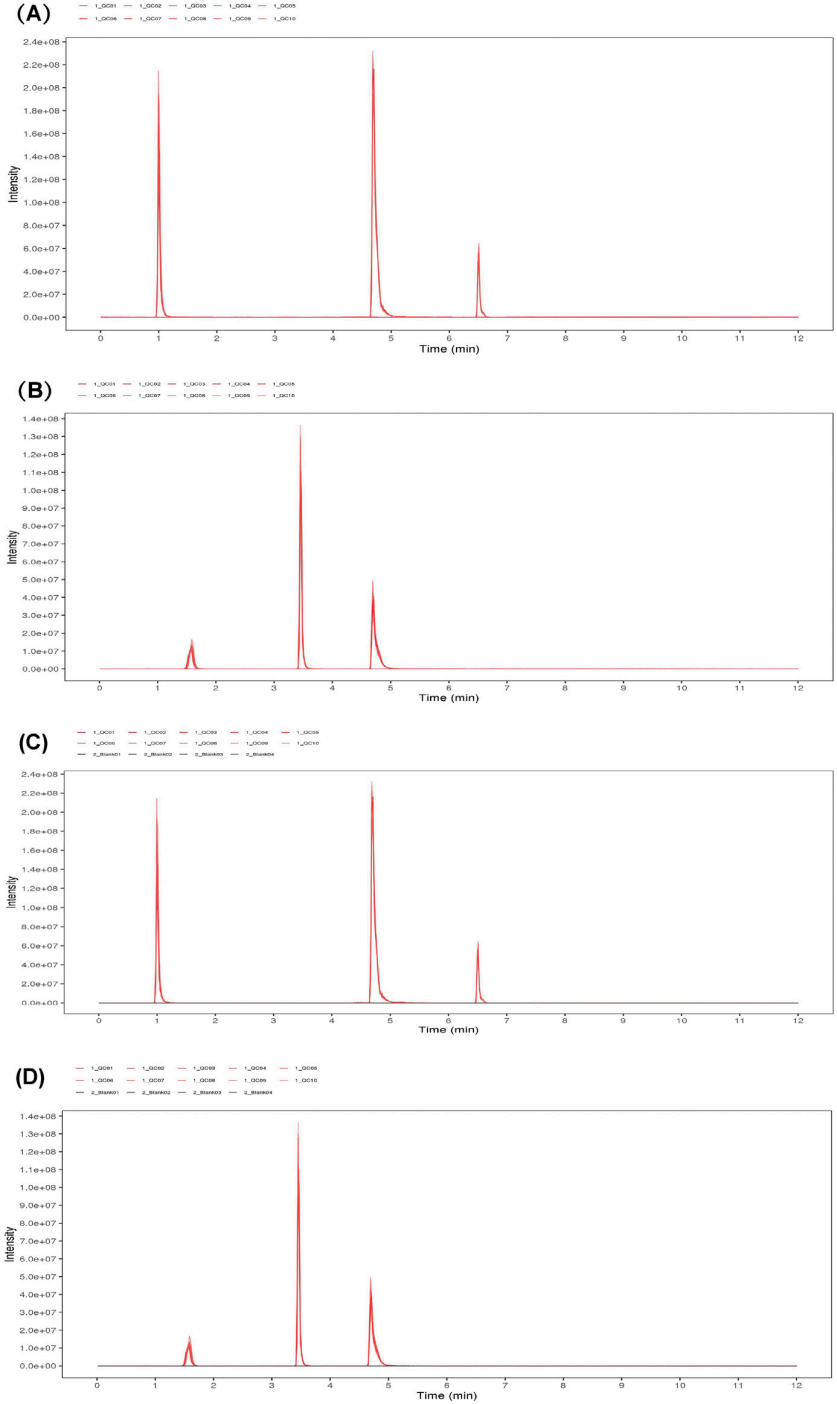
EIC plots of internal standard positive **(A)** and negative **(B)** ions in QC samples. EIC plots of positive **(C)** and negative **(D)** ions of internal standards in QC samples.

**FIGURE 8:**
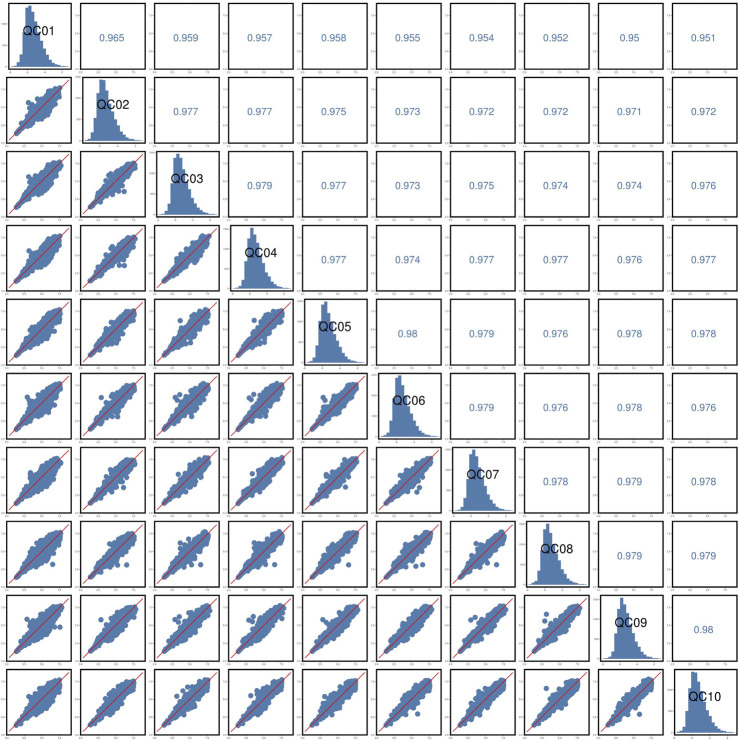
QC samples correlation analysis.

The response peak of the QC sample internal standard was analyzed first (Figure 7). Results indicated that the retention time and response intensity of the QC sample are highly stable. No significant peaks of the internal standard were detected in the blank sample, demonstrating stable instrument data collection and effective control of material residues and cross-contamination between samples. Subsequently, a correlation analysis of the QC samples was conducted (Figure 8), revealing a correlation coefficient close to 1, which indicates a high degree of correlation. This suggests that the experimental data quality is reliable. Overall, the robustness of the LC-MS method was confirmed.

### 3.5 PCA analysis

**FIGURE 9:**
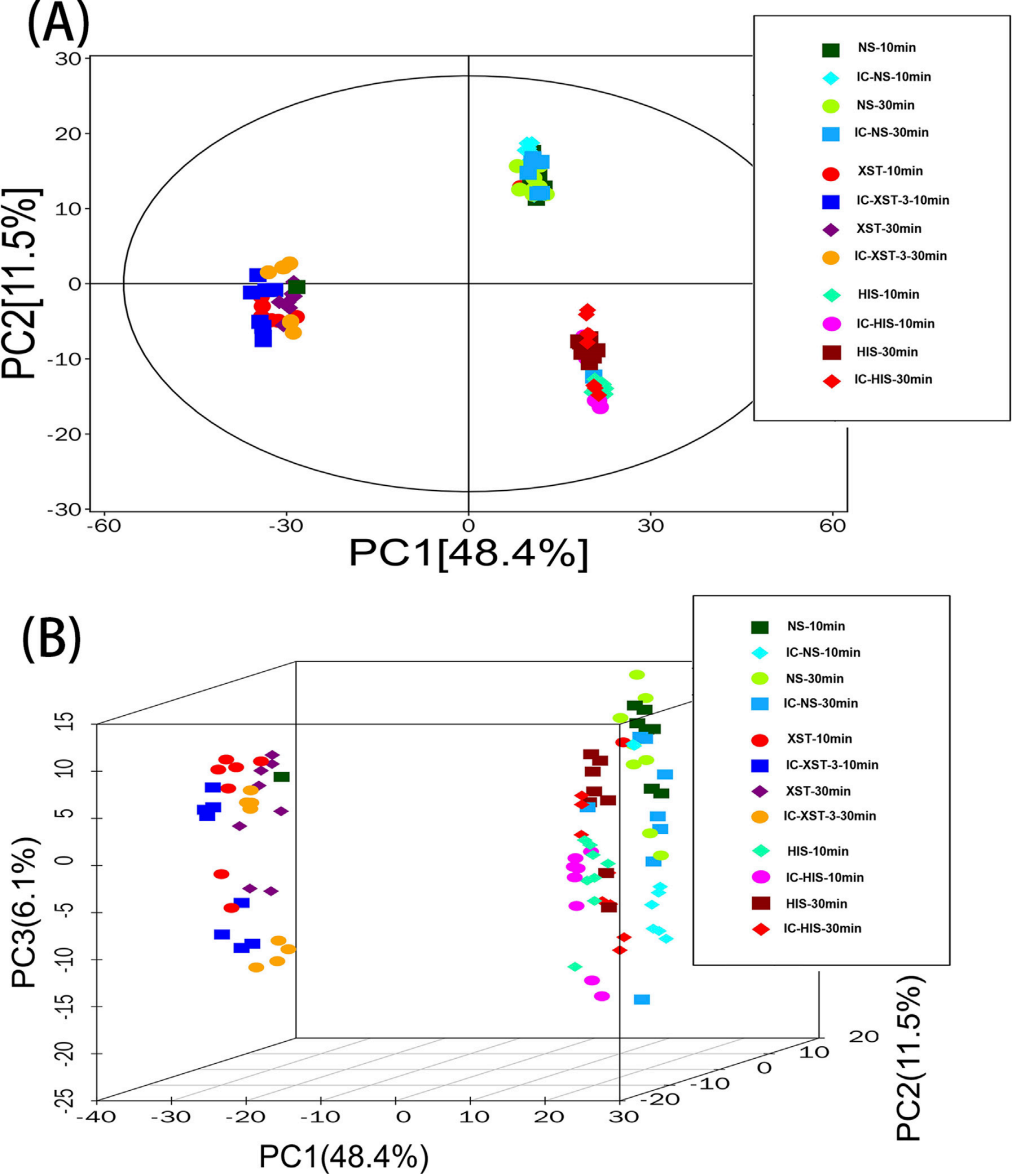
PCA score plot of total sample. **(A)**: 2D plot. **(B)**: 3D plot.

PCA is an unsupervised data analysis technique (Gao et al., 2024) used here to assess intergroup differences. In the diagram (Figure 9), the horizontal and vertical axes represent the scores of the first two principal components. Each scatter point denotes a sample, with points of the same color representing samples from the same group. The proximity of sample points suggests similarity in metabolite types and contents, while greater distances between points indicate larger differences in metabolic profiles. The drug treatment groups predominantly fall within the 95% confidence interval and are distinguishable from the non-drug group.

### 3.6 OPLS-DA analysis

**FIGURE 10:**
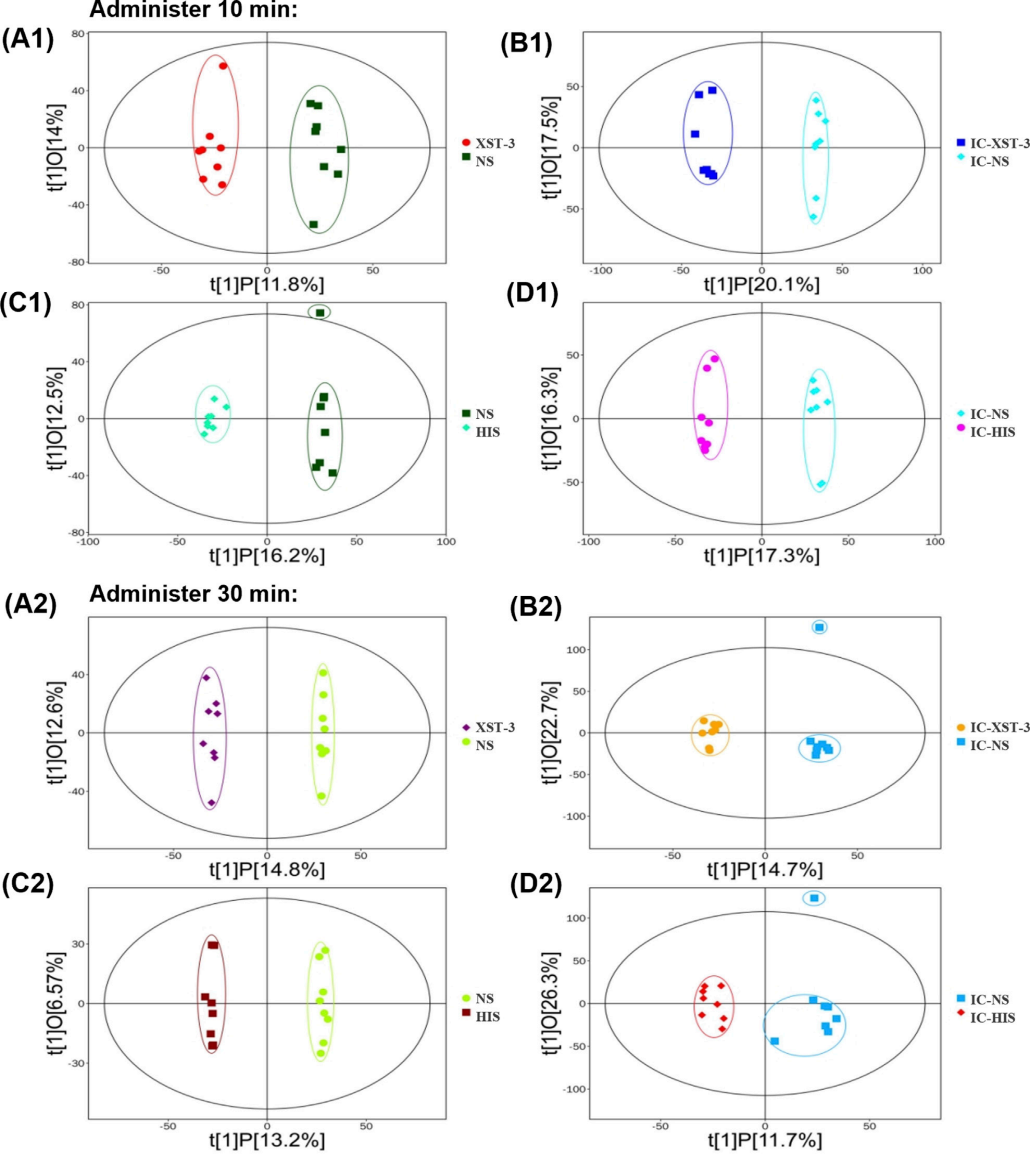
OPLS-DA score plot. **(A1, A2)**: Comparison of XST-3 group and NS group. **(B1, B2)**: Comparison of IC-XST-3 group and IC-NS group. **(C1, C2)**: Comparison of NS group and HIS group. **(D1, D2)**: Comparison of IC-NS group and IC-HIS group.

OPLS-DA is a supervised classification model. First, we validated the model’s effectiveness using 7-fold cross-validation, followed by a permutation test for further assessment. R^2^Y represents the model’s explanatory ability, while Q^2^ indicates its predictive power. The closer these values are to 1, the better the model’s fit. As shown in figure (Figure 10), the R^2^Y for each group exceeds 0.9, Q^2^ exceeds 0.4, and the Q^2^ intercept on the ordinate is below 0, indicating that the model is not overfitted and the groups are completely separable.

### 3.7 Identification of differential metabolites

Potential differential metabolites (*p* < 0.05, VIP >1) were identified through multivariate statistical analysis using databases such as HMDB and KEGG. Endogenous differential metabolites were further validated by assessing their pathway impacts and consulting the relevant literature. As a result, 10 differential metabolites were identified in the XST group (Table 4) and IC-XST-3 group (Table 5) respectively, while 21 and 20 differential metabolites were found in the HIS group (Table 6) and IC-HIS group (Table 7), respectively.

**TABLE 4:** Differential metabolites in XST-3 group.

Time	MS2 name	Formula	Reaction time (s)	m/z	HMDB	FC	Pathway
10 min	Diethanolamine	C_4_H_11_NO_2_	326.79	106.09	HMDB0004437	1.84	Glycerophospholipid metabolism
	6-Keto-prostaglandin F1α	C_20_H_34_O_6_	171.14	369.23	HMDB0002886	2.32	Arachidonic acid metabolism
	^a^Deoxycholic acid	C_24_H_40_O_4_	172.83	410.33	HMDB0000626	0.10	Bile secretion
	^a^Dihydrotestosterone	C_19_H_30_O_2_	255.78	255.21	HMDB0002961	0.05	Steroid hormone biosynthesis
	Pyroglutamic acid	C_5_H_7_NO_3_	313.31	128.03	HMDB0000267	1.19	Steroid hormone biosynthesis
	^a^Ursodeoxycholic acid	C_24_H_40_O_4_	167.38	391.28	HMDB0000946	0.11	Secondary bile acid biosynthesis
	Cholic acid	C_24_H_40_O_5_	203.51	407.28	HMDB0000619	0.07	Bile secretion
	Biotin	C_10_H_16_N_2_O_3_S	133.08	265.06	HMDB0000030	0.45	Biotin metabolism
30 min	N1-Methyl-2-pyridone-5-carboxamide	C_7_H_8_N_2_O_2_	84.33	153.07	HMDB0004193	1.54	Nicotinate and nicotinamide metabolism
	N-Acetylornithine	C_7_H_14_N_2_O_3_	324.63	187.07	HMDB0003357	0.57	Arginine Biosynthesis
	^a^Deoxycholic acid	C_24_H_40_O_4_	172.83	410.33	HMDB0000626	0.12	Bile secretion
	^a^Dihydrotestosterone	C_19_H_30_O_2_	255.78	255.21	HMDB0002961	0.06	Steroid hormone biosynthesis
	^a^Ursodeoxycholic acid	C_24_H_40_O_4_	167.38	391.28	HMDB0000946	0.15	Secondary bile acid biosynthesis

^a^ The same differential metabolites were present at 10 and 30 min postdose.

**TABLE 5:** Differential metabolites in IC-XST-3 group.

Time	MS2 name	Formula	Reaction time (s)	m/z	HMDB	FC	Pathway
10 min	Glycerophosphocholine	C_8_H_20_NO_6_P	402.94	258.11	HMDB0000086	1.33	Glycerophospholipid metabolism
	Indoleacetaldehyde	C_10_H_9_NO	32.88	158.06	HMDB0001190	1.29	Tryptophan metabolism
	1-Pyrroline-2-carboxylic acid	C_5_H_7_NO_2_	360.30	114.06	HMDB0006875	0.70	Arginine and proline metabolism
	Glyceric acid	C_3_H_6_O_4_	317.36	105.02	HMDB0000139	0.80	Glycerolipid metabolism
	N-Acetylornithine	C_7_H_14_N_2_O_3_	324.63	187.07	HMDB0003357	1.60	Arginine biosynthesis
	Glycerol	C_3_H_8_O_3_	99.79	91.04	HMDB0000131	1.29	Glycerolipid metabolism
	^a^Dihydrotestosterone	C_19_H_30_O_2_	255.78	255.21	HMDB0002961	0.01	Steroid hormone biosynthesis
30 min	L-Carnitine	C_7_H_15_NO_3_	369.70	162.11	HMDB0000062	1.22	bile secretion
	^a^Dihydrotestosterone	C_19_H_30_O_2_	255.78	255.21	HMDB0002961	0.03	Steroid hormone biosynthesis
	Cholic acid	C_24_H_40_O_5_	203.51	407.28	HMDB0000619	0.26	bile secretion
	5,6-DHET	C_20_H_34_O_4_	81.71	337.24	HMDB0002343	2.01	Arachidonic acid metabolism
	Imidazole-4-acetaldehyde	C_5_H_6_N_2_O	151.17	111.06	HMDB0003905	1.24	Histidine metabolism

^a^ The same differential metabolites were present at 10 and 30 min postdose.

**TABLE 6:** Differential metabolites in HIS group.

Time	MS2 name	Formula	Reaction time (s)	m/z	HMDB	FC	Pathway
10 min	Taurine	C_2_H_7_NO_3_S	311.95	126.02	HMDB0000251	0.73	Primary bile acid biosynthesis
	^a^Histamine	C_5_H_9_N_3_	405.19	112.09	HMDB0000870	0.01	Histidine metabolism
	Succinic acid	C_4_H_6_O_4_	395.72	117.02	HMDB0000254	0.43	Nicotinate and nicotinamide metabolism
	^a^1-Methylhistamine	C_6_H_11_N_3_	367.39	126.10	HMDB0000898	0.13	Histidine metabolism
	^a^Glycerophosphocholine	C_8_H_20_NO_6_P	402.94	258.11	HMDB0000086	3.44	Glycerophospholipid metabolism
	L-Carnitine	C_7_H_15_NO_3_	369.70	162.11	HMDB0000062	0.85	bile secretion
	Sphinganine	C_18_H_39_NO_2_	129.59	302.31	HMDB0000269	0.51	Sphingolipid metabolism
	Glyceric acid	C_3_H_6_O_4_	317.36	105.02	HMDB0000139	0.72	Glycerolipid metabolism
	Sphingosine	C_18_H_37_NO_2_	87.87	300.29	HMDB0000252	0.42	Sphingolipid metabolism
	^a^6-Keto-prostaglandin F1α	C_20_H_34_O_6_	171.14	369.23	HMDB0002886	0.49	Arachidonic acid metabolism
	4-Imidazolone-5-propionic acid	C_6_H_8_N_2_O_3_	360.30	157.06	HMDB0001014	0.57	Histidine metabolism
	Glycerol	C_3_H_8_O_3_	99.79	91.04	HMDB0000131	0.64	Glycerolipid metabolism
	15-Deoxy-d-12,14-PGJ2	C_20_H_28_O_3_	61.13	317.21	HMDB0005079	0.33	Arachidonic acid metabolism
	Biotin	C_10_H_16_N_2_O_3_S	133.08	265.06	HMDB0000030	0.51	Biotin metabolism
	Thromboxane B2	C_20_H_34_O_6_	186.57	369.23	HMDB0003252	0.57	Arachidonic acid metabolism
30 min	Propionic acid	C_3_H_6_O_2_	255.56	73.03	HMDB0000237	0.71	Nicotinate and nicotinamide metabolism
	^a^Histamine	C_5_H_9_N_3_	405.19	112.09	HMDB0000870	0.03	Histidine metabolism
	D-Proline	C_5_H_9_NO_2_	327.88	116.07	HMDB0003411	0.86	Arginine and proline metabolism
	Succinic acid semialdehyde	C_4_H_6_O_3_	97.50	101.02	HMDB0001259	0.63	Nicotinate and nicotinamide metabolism
	^a^1-Methylhistamine	C_6_H_11_N_3_	367.39	126.10	HMDB0000898	0.11	Histidine metabolism
	^a^Glycerophosphocholine	C_8_H_20_NO_6_P	402.94	258.11	HMDB0000086	2.50	Glycerophospholipid metabolism
	^a^6-Keto-prostaglandin F1α	C_20_H_34_O_6_	171.14	369.23	HMDB0002886	0.51	Arachidonic acid metabolism
	Arachidonic acid	C_20_H_32_O_2_	37.57	303.23	HMDB0001043	1.32	Arachidonic acid metabolism
	Sphingosine 1-phosphate	C_18_H_38_NO_5_P	307.17	380.26	HMDB0000277	2.22	Sphingolipid metabolism
	N4-Acetylaminobutanal	C_6_H_11_NO_2_	119.94	130.09	HMDB0004226	0.64	Arginine and proline metabolism

^a^ The same differential metabolites were present at 10 and 30 min postdose.

**TABLE 7:** Differential metabolites in IC-HIS group.

Time	MS2 name	Formula	Reaction time (s)	m/z	HMDB	FC	Pathway
10 min	^a^Histamine	C_5_H_9_N_3_	405.19	112.09	HMDB0000870	0.01	histamine metabolism
	^a^1-Methylhistamine	C_6_H_11_N_3_	367.39	126.10	HMDB0000898	0.15	Histidine metabolism
	^a^Glycerophosphocholine	C_8_H_20_NO_6_P	402.94	258.11	HMDB0000086	4.60	Glycerophospholipid metabolism
	Indoleacetaldehyde	C_10_H_9_NO	32.88	158.06	HMDB0001190	1.25	Tryptophan metabolism
	Hypotaurine	C_2_H_7_NO_2_S	359.75	108.01	HMDB0000965	0.69	Taurine and hypotaurine metabolism
	Formylanthranilic acid	C_8_H_7_NO_3_	84.57	164.03	HMDB0004089	0.76	Tryptophan metabolism
	1-Methylnicotinamide	C_7_H_9_N_2_O	324.46	137.07	HMDB0000699	0.68	bile secretion
	^a^Phosphocreatine	C4H10N3O5P	452.13	212.04	HMDB0001511	0.48	Arginine and proline metabolism
	Sphinganine	C_18_H_39_NO_2_	129.59	302.31	HMDB0000269	0.56	Sphingolipid metabolism
	N1-Methyl-2-pyridone-5-carboxamide	C_7_H_8_N_2_O_2_	84.33	153.07	HMDB0004193	0.68	Nicotinate and nicotinamide metabolism
	Arachidonic acid	C_20_H_32_O_2_	37.57	303.23	HMDB0001043	1.34	Arachidonic acid metabolism
	4-Imidazolone-5-propionic acid	C_6_H_8_N_2_O_3_	360.30	157.06	HMDB0001014	0.69	Histidine metabolism
	Glycerol	C_3_H_8_O_3_	99.79	91.04	HMDB0000131	0.75	Glycerolipid metabolism
	Prostaglandin F2α	C_20_H_34_O_5_	70.41	337.23	HMDB0001139	0.58	Arachidonic acid metabolism
	Methylimidazole acetaldehyde	C_6_H_8_N_2_O	7.41	125.07	HMDB0004181	2.16	Histidine metabolism
	Sphingosine 1-phosphate	C_18_H_38_NO_5_P	307.17	380.26	HMDB0000277	1.59	Sphingolipid metabolism
30 min	^a^Histamine	C_5_H_9_N_3_	405.19	112.09	HMDB0000870	0.15	histamine metabolism
	^a^1-Methylhistamine	C_6_H_11_N_3_	367.39	126.10	HMDB0000898	0.23	Histidine metabolism
	^a^Glycerophosphocholine	C_8_H_20_NO_6_P	402.94	258.11	HMDB0000086	3.84	Glycerophospholipid metabolism
	Cholesterol	C_27_H_46_O	31.17	369.35	HMDB0000067	0.64	bile secretion
	N-Acetylornithine	C_7_H_14_N_2_O_3_	373.25	175.11	HMDB0003357	1.48	Arginine biosynthesis
	^a^Prostaglandin F2α	C_20_H_34_O_5_	70.41	337.23	HMDB0001139	2.37	Arachidonic acid metabolism
	Tryptamine	C_10_H_12_N_2_	442.56	161.10	HMDB0000303	1.75	Tryptophan metabolism
	5,6-DHET	C_20_H_34_O_4_	81.71	337.24	HMDB0002343	1.78	Arachidonic acid metabolism

^a^ The same differential metabolites were present at 10 and 30 min postdose.

### 3.8 Metabolite pathway analysis

**FIGURE 11:**
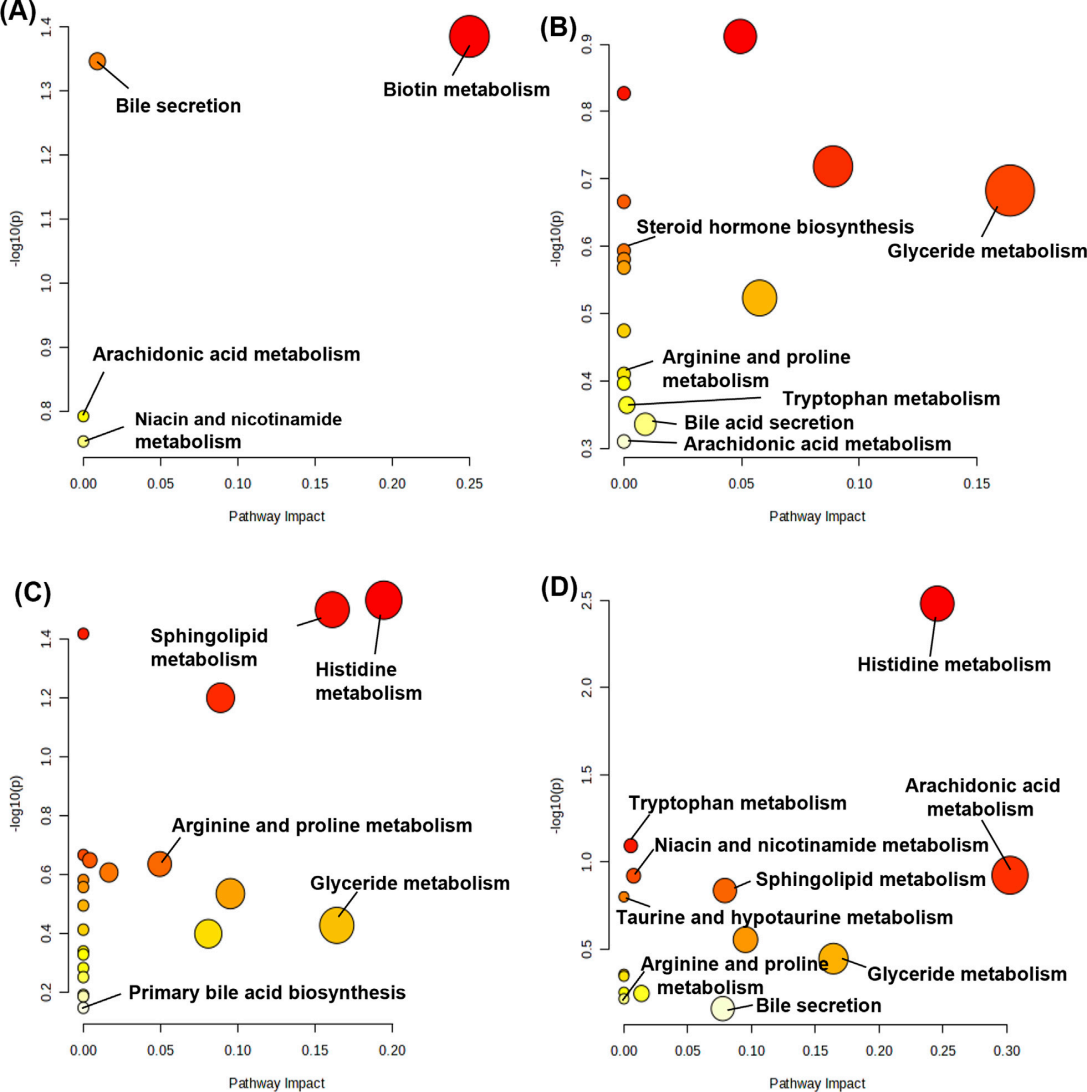
Metabolic pathway map. **(A)**: XST-3 group-related pathways. **(B)**: HIS group-related pathways. **(C)**: IC-XST-3 group-related pathways. **(D)**: IC-HIS group-related pathways.

**FIGURE 12:**
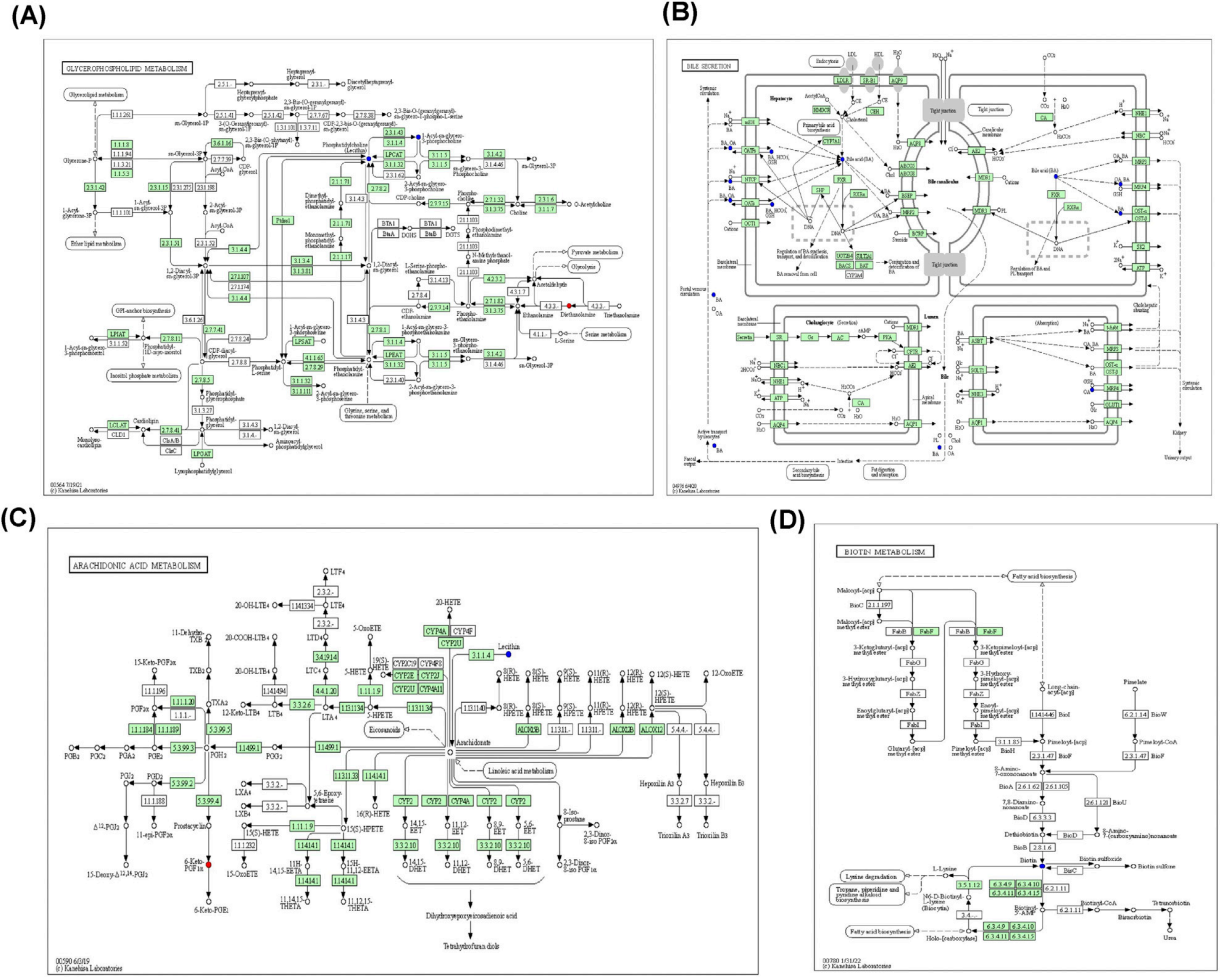
XST-3 key metabolic pathways. **(A)**: Glycerophospholipid metabolism. **(B)**: Bile secretion. **(C)**: Arachidonic acid metabolism. **(D)**: Biotin metabolism.

**FIGURE 13:**
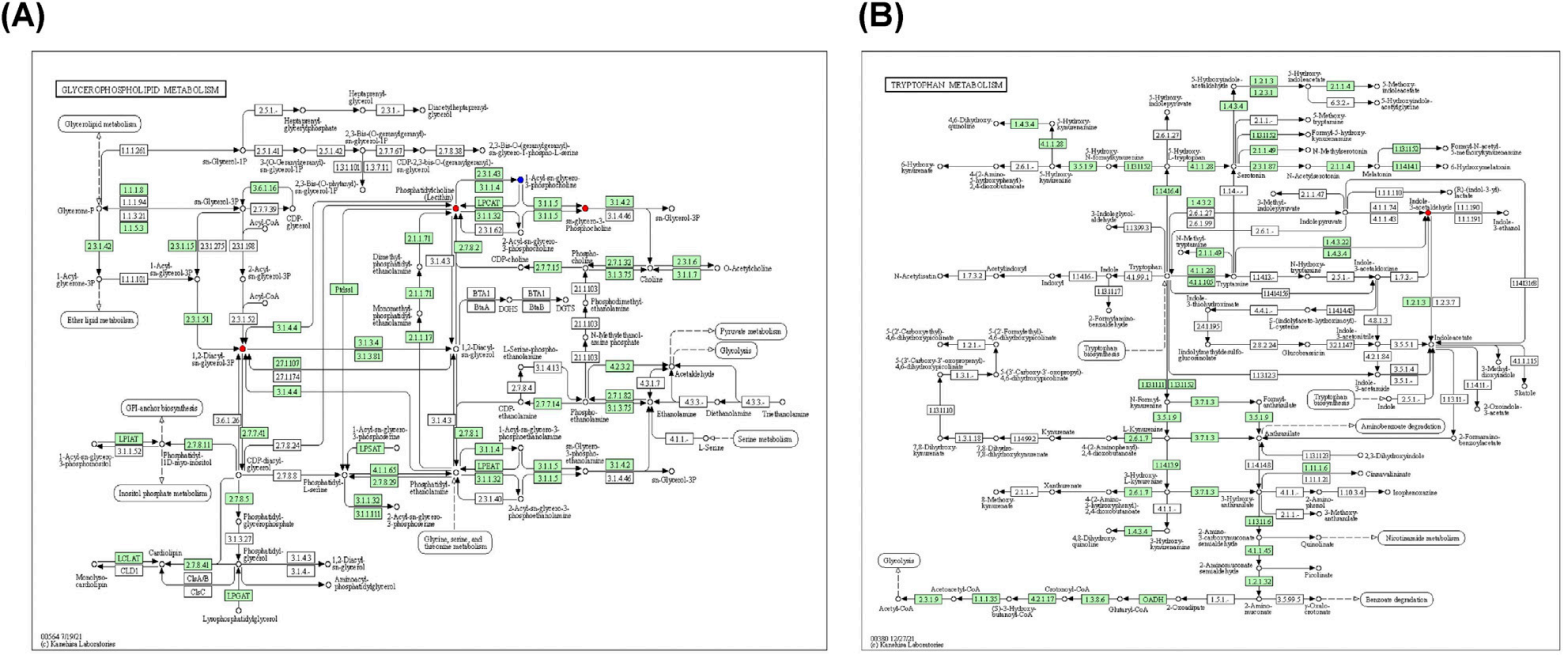
IC**-**XST-3 key metabolic pathways. **(A)**: Glycerophospholipid and glyceride metabolism. **(B)**: tryptophan metabolism.

Metabolic pathway maps for each group (Figure 11) were generated using the MetaboAnalyst analysis platform (http://new.metaboanalyst.ca/ModuleView.xhtml). In these maps, a higher *p*-value indicates greater relevance of the metabolic pathways to the drug effects. Analysis revealed that the XST-3 group’s pathways involve glycerophospholipid, arachidonic acid, bile secretion, and biotin metabolism. For the IC-XST-3 group, the pathways include glycerophospholipid, tryptophan, and glyceride metabolism. The HIS group’s pathways encompass histamine, arachidonic acid, and sphingolipid metabolism. The metabolic pathways for the IC-HIS group include histamine, glycerophospholipid, and tryptophan metabolism. In addition, the key metabolic pathways for the XSTI treatment groups were illustrated in detail (Figures 12, 13). In this map, circular nodes represent metabolites, with red indicating significantly upregulated metabolites and blue indicating significantly downregulated metabolites. Rectangular boxes represent genes (proteins) involved in the pathway, and lines indicate the direction of metabolic reactions.

## 4 Discussion

TCMI exhibits a rapid onset of action and high bioavailability compared to other dosage forms (Guo, X. et al., 2012). However, adverse reactions induced by TCMI, particularly ARs, have hindered its clinical application (Li, L. et al., 2012).

While prior research has focused primarily on drug-related factors, the disease-syndrome-based toxicology concept proposed by Xiao (Bai et al., 2017) underscores the importance of considering individual specificity, such as immune and metabolic abnormalities, in adverse reaction studies (Ulrich, 2007). Individual specificity, encompassing immune and metabolic abnormalities, is a key factor in drug-specific injury. Skin and liver are common targets of drug-specific injury, leading to clinical manifestations such as liver damage and various allergic diseases (Mosedale and Watkins, 2020). Studies have indicated that specific individuals are more susceptible to allergic diseases (Verma et al., 2018), underscoring the role of individual constitutions in the occurrence of such conditions.

In this experiment, we investigated the differences in ARs induced by XSTI in two different body states: normal and immunocompromised. Auricular blue staining and EB exudation experiments demonstrated reduced sensitivity to high doses of XSTI in immunocompromised mice. Notably, at low doses, immunocompromised mice exhibited larger auricular blue staining areas and increased EB exudation compared to normal mice, contrasting with results at the high doses. Since the behavioral results indicated that ARs induced by XSTI at low doses were negative, this opposite result may stem from metabolic changes in immunosuppressive mice. For instance, N1-methyl-2-pyridone-5-carboxamide, a final metabolite of niacin known to enhance endothelial function, vascular inflammatory responses, and angiogenesis (Pang et al., 2016), was downregulated in immunocompromised mice and upregulated in normal mice. This suggests that metabolic alterations may affect vascular permeability in immunocompromised mice, contributing to the opposite results observed at low doses.

In the ELISA experiment, TPS, β-Hex, and HIS, inflammatory mediators released through mast cell degranulation (Roy et al., 2021), were used to evaluate the occurrence of ARs, while IgE was employed to evaluate the association between XSTI and type I hypersensitivity. Surprisingly, IgE levels in the XST-3 group were significantly elevated compared to the NS group (*p* < 0.01), suggesting that XSTI may mediate allergic reactions through the IgE pathway. However, the reactions were observed to occur within 30 min of the first dose, confirming that ARs remain the primary response.

We hypothesized metabolic pathways of ARs through differential metabolites. The rapid onset of ARs may be linked to biotin metabolism, observed within 30 min after administration. Dihydrotestosterone, a metabolite that appeared exclusively in XSTI-treated groups and exhibited a decreasing trend, warrants further investigation as a potential biomarker for ARs. Additionally, studying other metabolites and related enzymes may provide valuable insights and serve as a foundation for future research on ARs induced by XSTI. Furthermore, the variations in metabolic pathways between normal and immunocompromised mice indicate that the body’s immune status can influence the sensitizing effect of TCMI, thereby revealing the immune specificity of ARs.

### 4.1 Biotin metabolism

Biotin regulates immune and inflammatory functions by modulating lymphocytes and transcription factors, with its deficiency being linked to skin inflammation and immune dysfunction (Seki, 2006; Kuroishi, 2015). In this study, biotin metabolism was observed in the normal groups. In the XST-3 and HIS groups, biotin levels were downregulated at 10 min and recovered by 30 min, suggesting that XSTI and HIS may trigger inflammatory responses by suppressing biotin metabolism. Notably, the timing of biotin metabolism aligns with the rapid onset of ARs, further supporting its involvement in ARs regulation.

### 4.2 Bile secretion and primary and secondary bile acid biosynthesis

Bile acids play a pivotal role in regulating inflammation by inhibiting the Nlrp3 inflammasome (Guo et al., 2016) and activating the farnesoid X and G protein-coupled bile acid receptors, which are associated with the regulation of monocyte and macrophage functions (Fiorucci et al., 2020). In the XST-3 group, deoxycholic acid, cholic acid, and ursodeoxycholic acid were downregulated. In the IC-XST-3 group, cholic acid showed a downward trend, while L-carnitine exhibited an upward trend. The HIS group displayed downregulation of taurine and L-carnitine. In the IC-HIS group, 1-methylnicotinamide and cholesterol were downregulated. Cholesterol serves as the primary precursor for bile acid synthesis, while taurine enhances bile acid solubility through conjugation. Cholic acid, deoxycholic acid, and ursodeoxycholic acid are major components of primary and secondary bile acids (Di Ciaula et al., 2017). The marked downregulation of these metabolites indicates that XSTI and HIS suppress the bile acid synthesis pathway, leading to ARs.

### 4.3 Arachidonic acid metabolism

Arachidonic acid (AA), a key component of cell membrane lipids, is primarily metabolized by cyclooxygenase (COX), lipoxygenase, and cytochrome P450 (CYP450) enzymes. Its metabolites, including prostaglandins, thromboxanes, leukotrienes, and hydroxyeicosatetraenoic acids, trigger various inflammatory responses (Wang et al., 2019).

In our study, 6-keto-PGF1α was upregulated in the XST-3 group, and 5,6-dihydroxyeicosatrienoic acid (5,6-DHET) was upregulated in the IC-XST-3 group. In the HIS group, 6-keto-PGFα, 15-deoxy-d-12,14-PGJ2, and thromboxane B2 were downregulated, while arachidonic acid was upregulated. In the IC-HIS group, arachidonic acid and 5,6-DHET were upregulated, and prostaglandin F2α was downregulated at 10 min and upregulated at 30 min. In the XSTI treatment groups, 6-keto-PGF1α and 5,6-DHET were significantly upregulated. These are degradation products of AA metabolized through the COX pathway into PGI2 and EET, respectively, which can dilate blood vessels and reduce platelet aggregation (Levi-Rosenzvig et al., 2017). This hypothesized that XSTI induces ARs by upregulating the COX metabolic pathway of AA. In the HIS treatment groups, most AA metabolites were downregulated, while AA levels increased, suggesting that HIS inhibits AA metabolism, leading to AA accumulation.

### 4.4 Niacin and nicotinamide metabolism

Niacin, a precursor of nicotinamide adenine dinucleotide (NAD^+^) and nicotinamide adenine dinucleotide phosphate (NADP^+^) (Gasperi et al., 2019), can inhibit vascular inflammation (Kamanna and Kashyap, 2008) and alleviate systemic inflammatory responses by reducing inflammatory factors produced by monocytes and macrophages (Siavashani et al., 2020). Excessive niacin, however, may lead to skin vasodilation and flushing (Javaid and Mudavath, 2024).

In this study, N1-methyl-2-pyridone-5-carboxamide (2PY) was upregulated in the XST-3 group, while succinate, propionate, and succinate semialdehyde were downregulated in the HIS group, and 2PY was downregulated in the IC-HIS group. Succinate is an intermediate of the Krebs cycle, which is linked to metabolic regulation and inflammation and requires NAD⁺/NADP⁺ as essential cofactors for its proper functioning (Tang et al., 2022). In the HIS-treated groups, the terminal niacin metabolite 2PY and succinate-related metabolites were downregulated, suggesting that HIS inhibits niacin metabolism, thereby promoting inflammation. This inhibition likely reduces NAD⁺/NADP⁺ synthesis, impairing the Krebs cycle. Conversely, 2PY was upregulated in the XST-3 group, indicating that XSTI may regulate ARs by enhancing nicotinamide metabolism.

### 4.5 Tryptophan metabolism

Tryptophan can be converted into niacin, and its metabolism primarily occurs via the kynurenine, serotonin, and indole pathways, playing roles in regulating inflammation, metabolism, immune responses, and neural functions (Xue et al., 2023). In the IC-XST-3 group, indole acetaldehyde was upregulated. In the IC-HIS group, indole acetaldehyde and tryptamine were upregulated, while formyl anthranilic acid was downregulated. Differential metabolites were observed exclusively in the immunocompromised groups, primarily involving the indole metabolic pathway. In this pathway, tryptophan is converted into tryptamine and indole derivatives under the influence of gut microbiota (Xue et al., 2023). Indole derivatives contribute to maintaining immune system homeostasis and activate the aryl hydrocarbon receptor, which regulates immune cells and gut barrier integrity (Li, 2023). It is hypothesized that XSTI and HIS modulate inflammatory responses in immunocompromised mice through the regulation of tryptophan metabolism.

### 4.6 Steroid hormone biosynthesis

Steroid hormones, including sex hormones and adrenal cortical hormones, exert immunosuppressive and anti-inflammatory effects in the body (Wang, C. et al., 2015). In the XST-3 group, pyroglutamic acid was upregulated, while dihydrotestosterone (DHT) was downregulated. Similarly, DHT was downregulated in the IC-XST-3 group. DHT exerts multiple inhibitory effects on innate and adaptive immune responses, with its deficiency associated with enhanced expression of inflammatory factors (Ikuta et al., 2022). The downregulation of DHT in the XSTI-treated groups suggests that XSTI may promote inflammation by suppressing DHT levels and inducing ARs. Moreover, DHT could serve as a potential biomarker for XSTI-induced ARs.

### 4.7 Lipid metabolism

Lipid metabolism involves glycerolipid, glycerophospholipid, and sphingolipid pathways, which work synergistically to maintain cellular functions by supporting membrane structure and signal transduction (Ristovski et al., 2021). In the XST-3 group, diethanolamine levels were upregulated. Glycerophosphocholine and glycerol were upregulated in the IC-XST-3 group, while glyceric acid was downregulated. The HIS group showed upregulation of glycerophosphatidic acid choline and sphingosine 1-phosphate (S1P), alongside downregulation of sphingosine, glyceric acid, and glycerol. In the IC-HIS group, glycerophosphocholine and S1P were upregulated, whereas sphingosine and glycerol were downregulated. Glycerophospholipids activate calcium signaling and PKC signaling through hydrolysis by PLC, which induces MC degranulation (Yang, Y. et al., 2019). S1P is a bioactive intermediate in sphingolipid metabolism, activates the RhoA/ROCK pathway, regulates the actin cytoskeleton, increases vascular permeability, and triggers inflammatory responses (Piao et al., 2024). These findings suggest that XSTI primarily regulates glycerophospholipid metabolism, while HIS impacts both glycerophospholipid and sphingolipid metabolism, acting on MC to induce degranulation and triggering ARs.

### 4.8 Arginine and proline metabolism

Arginine metabolism is crucial for immune cell responses, with its metabolites playing key roles in T cell activation and the regulation of both innate and adaptive immunity (Li, S. et al., 2022). Proline is vital for protein synthesis, wound healing, antioxidant defense, and immune function (Wu et al., 2011). Arginine and proline interconvert via ornithine as an intermediate to sustain physiological metabolic functions (Wang et al., 2024).

N-acetylornithine was downregulated in the XST-3 group. In the IC-XST-3 group, 1-pyrroline-2-carboxylic acid showed a downward trend, while N-acetylornithine was upregulated. D-proline and N4-acetamide butyraldehyde were downregulated in the HIS group. In normal groups, the downregulation of metabolites suggests that the drugs may weaken T cell and macrophage functions and disrupt the immune balance by inhibiting arginine and proline metabolism. Conversely, the upregulation of N-acetylornithine in the IC-XST-3 group reflected the body’s attempt to restore immune function through compensatory metabolic adjustments.

## 5 Conclusion

In this study, we used XSTI as a model drug to explore the relationship between AR intensity, TCMIs, and immune status. The results demonstrated that XSTI induces ARs in a dose-dependent manner and that the body’s immune status affects the severity of ARs, with reduced intensity under the immunosuppressive state. Metabolomic analysis revealed that XSTI modulates ARs by influencing several metabolic pathways involved in the regulation of cellular membrane phospholipids, MC functionality, and inflammatory responses. The differential metabolite dihydrotestosterone, which appeared only in the XSTI-treated group, has the potential to serve as a biomarker for the onset of XSTI-induced ARs. In immunocompromised mice, the attenuation of ARs may be due to the inhibition of inflammatory cytokine release via tryptophan metabolism. Differences in metabolic pathways related to varying immune statuses likely contribute to the decreased AR intensity observed in immunocompromised mice. However, certain limitations exist in this study. The investigation focused on the effects of immunosuppression on ARs, leaving immune stress unaddressed. Further research on ARs under immune stress conditions is required to comprehensively elucidate the relationship between ARs and the body’s immune status. Although metabolomics analysis identified metabolic pathways involved in XSTI-induced ARs, these pathways were not validated. Future studies should incorporate experimental validation to uncover the mechanisms of these pathways and clarify their connection to immune status and ARs.

## Data Availability

The raw data supporting the conclusions of this article will be made available by the authors, without undue reservation.
